# Selection of indicators for tonsillectomy in adults with recurrent tonsillitis

**DOI:** 10.1186/1472-6815-5-7

**Published:** 2005-09-13

**Authors:** Priit Kasenõmm, Andres Piirsoo, Mart Kull, Mart Kull, Marika Mikelsaar

**Affiliations:** 1 Department of Microbiology, Tartu University, Ravila St. 19, Tartu 50411, Estonia; 2 Department of Otorhinolaryngology, Tartu University Clinicum, Kuperjanovi St. 1, Tartu 51003, Estonia; 3 Department of General and Molecular Pathology, Medical Faculty, Tartu University, Ravila St. 19, Tartu 50411, Estonia

## Abstract

**Background:**

We aimed to find some new indicators for tonsillectomy (TE) in adults with recurrent tonsillitis (RT) by exploring whether the frequency of tonsillitis episodes and the length of morbidity period are associated with the macroscopic signs of sclerotic process in tonsils and microbiological data assessed by culture, molecular (PCR) and transmission electron microscopy (EM) methods.

**Methods:**

The study involved 62 RT patients admitted for TE (age range 15–35, median 22 years) and 54 healthy volunteers (age range 18–24, median 20 years). The index of tonsillitis (IT) was calculated by multiplying the number of tonsillitis episodes per year by the morbidity period in years. On oropharyngeal examination the presence or absence of three sclerotic signs was evaluated: tonsillar sclerosis, obstruction of tonsillar crypts and scar tissue on the tonsils. The occurrence of *Streptococcus pyogenes *was assessed by culture and PCR methods in 24 tonsillar core specimens. The samples for EM investigation of crypt epithelium were taken from 10 removed tonsils.

**Results:**

The IT values were in positive correlation with the number of sclerotic signs on oropharyngeal examination (r = 0.325, P = 0.010). Based on the IT values and the presence or absence of tonsillar sclerosis and obstruction of tonsillar crypts the receiver-operating curve (ROC) was constructed. It revealed that an IT score of 36 is an optimal cut-off value for prediction of sclerotic type tonsils. *S. pyogenes *was never found by culture, but its presence by PCR in nearly one third (29%) of diseased tonsillar tissue specimens was tightly associated with longer morbidity. EM revealed coccoid forms of intracellular bacteria in the crypt epithelium, which was accompanied with the damage of tight junctions between epithelial cells.

**Conclusion:**

The index of tonsillitis ≥36, being a combination between the frequency of tonsillitis and the length of morbidity period, predicts the sclerotic process in recurrently inflamed tonsils. Therefore, the high IT values could serve as an indicator for TE in adults. The correlation between the longer morbidity period and the presence of *S. pyogenes *by PCR suggests that persistent infection may have a role in maintenance of recurrent inflammation in tonsils.

## Background

Recurrent tonsillitis (RT) is a chronic inflammatory process in palatine tonsils. A leading therapeutic approach for such a condition has been tonsillectomy (TE). Traditionally, recommendation for TE has depended primarily on the frequency of tonsillitis episodes. Patients with at least three episodes per year, despite adequate medical therapy, may be considered as candidates for TE, and surgical treatment is definitely recommended for patients with more than four or five episodes per year [1,2]. Adult patients often have fewer or less severe tonsillitis episodes, yet the dominance of other indices of chronic disease, such as poor general health, time loss from school or work, decreased life quality due to systemic effects or comorbid diseases, group A β-hemolytic streptococcus carriage state, and increased serum concentrations of antibodies, which have also been considered as appropriate indicators for TE [3-5]. Unfortunately, there is no consensus for these arbitrarily used criteria, hinting at a need for more precise indicators.

Palatine tonsils are a part of the mucosa-associated lymphatic tissue (MALT), a specialized compartment of the immune system that serves as a first line of defence against environmental harmful factors, including pathogenic microbes [6]. Paradoxically, palatine tonsils themselves are quite frequently affected by bacterial and viral infections causing local inflammation and systemic reactions. Recurrent or chronic inflammation in the tonsillar tissue results in obstruction of tonsillar crypts due to tissue fibrosis, accompanied by distension of the crypts' bottom and retention of its content [7,8]. In our previous study, the sclerotic and inflammatory type tonsils were discriminated, based on the presence or absence of tonsillar sclerosis, obstruction of tonsillar crypts and scar tissue on the tonsils. The sclerotic process in tonsils was further evidenced by increased collagen content. It revealed that sclerotic type tonsils had remarkably lower count of neutrophils in its tissue, which increased the risk for bacteraemia during tonsillectomy [9]. Thus, extensive tissue fibrosis seemed to be a critical point in the RT pathogenesis where the defensive function of tonsils becomes impaired. Unfortunately, there are no studies showing whether the frequency of tonsillitis episodes and the length of morbidity period have an association with sclerotic process in recurrently inflamed tonsils. We suggest that such an approach might be helpful in finding some new indicators for TE.

Despite the high frequency in population, the etiology of RT has remained unclear. The surface and deep bacterial flora of recurrently inflamed tonsils consist of an abundance of potentially pathogenic aerobic and anaerobic bacteria [10-14]. Surprisingly, the isolation rate of *Streptococcus pyogenes *from adults with RT, the most important pathogen in acute tonsillar infection, is lower by conventional culture methods [15-17]. As the pathogenesis of various infectious diseases has been attributed to intracellularly residing bacteria, applying some modern methods could provide advantages to determine the occurrence of *S. pyogenes *and its role in the pathogenesis of RT.

The aim of this study was to assess whether the frequency of tonsillitis episodes and the length of morbidity period are associated with the macroscopic signs of sclerotic process in tonsils and microbiological data, assessed by culture, molecular (PCR) and transmission electron microscopy (EM) methods in RT patients admitted for TE (RT-TE).

## Methods

### Clinical cohort and follow-up

#### Patients

The study involved 62 RT-TE patients (age range 15–35, median 22 years; 41 females and 21 males) selected among 486 adults referred for TE due to recurrent attacks of tonsillitis episodes between October to December 2000, March to June and September to December 2001 at the Department of Otorhinolaryngology, Tartu University Clinicum. Every third patient (≥15 years of age) was selected from the operation list on two particular days of the week. Each patient had a history of recurrent tonsillitis episodes for at least one year, characterized by sore throat or swollen painful tonsils with fever or symptoms of systemic illness during exacerbations, but the absence of symptoms of possible viral upper respiratory tract infection, such as running nose and cough. As routinely throat cultures were not taken from adults with RT during each exacerbation, the episodes were considered of unknown cause. All patients had been referred for TE by an ENT surgeon from the Department of Otorhinolaryngology. The particular number of tonsillitis episodes per year was not set as an inclusion criterion in the present study. The exclusion criteria were the acute tonsillitis exacerbation, acute respiratory infection, and antibiotic therapy within the two previous months.

#### Control subjects

The control group consisted of 54 volunteer students (age range 18–24, median 20 years; 36 female and 18 male) who were not suffering from recurrent tonsillitis episodes. The study had approval from the Tartu University Research Ethics Committee, and in each case written informed consent was obtained from each participant.

#### Collection of history data and oropharyngeal examination

In RT-TE patients, the disease history data such as the number of tonsillitis episodes per year, the length of morbidity period in years, presence of documented comorbid diseases, usage of antibiotics and changes in life quality due to tonsillitis episodes were collected by one examiner (MK Jr), and the oropharyngeal examinations were performed by another (PK), who was blinded to the type of patients seen. In healthy controls, the same examiner conducted the oropharyngeal examinations separately. On oropharyngeal examination the presence or absence of the three characteristic signs of sclerotic process was evaluated: tonsillar sclerosis, obstruction of tonsillar crypts, and scar tissue on the tonsils. Tonsillar sclerosis was defined as increased tightness of tonsillar and peritonsillar tissue together with the fixation of palatine tonsil in the tonsillar fossa. The obstruction of the tonsillar crypts was documented when narrowing of the crypts' mouth was observed resulting in loss of clear cryptic pattern of the tonsillar surface. The scar tissue on the tonsils was defined as white tissue-spots or streaks on the tonsillar surface.

### Analysis of *S. pyogenes* occurrence in core tonsils

#### Bacteriological analysis

The occurrence of *S. pyogenes *in tonsillar core tissue was assessed in the first 24 tonsils removed from RT-TE patients. The bacteriological culture was performed according to the previously described method [18]. Briefly, after excision, one of the tonsils was placed in a sterile Petri dish and taken immediately to the microbiology laboratory. For a tonsillar core culture, approximately 0.2 g of tissue was aseptically excised and homogenized in a sterile mortar with a known amount of pre-reduced phosphate-buffered saline (PBS; pH 7.2) in the anaerobic glove box (Sheldon Manufacturing Inc., USA, with a gas mixture: 5% CO_2_, 5% H_2_, 90% N_2_) and was further serially diluted (10^-2 ^– 10^-7^). The dilutions were seeded on Columbia horse blood agar plates enriched with streptococcus selective supplement (Oxoid Ldt., UK). All plates were incubated for 48 hours at 36°C in an atmosphere enriched with 10% CO_2 _in Jouan IG150 incubator (Jouan, France). The culture plates were examined for the growth of β-hemolytic streptococci and selected colonies were Gram stained and subjected to microscopy. The β-hemolytic streptococci were distinguished from α-hemolytic streptococci by the type of hemolysis and were grouped using streptococcus latex agglutination test (Oxoid Ltd., UK).

#### PCR amplification

For molecular detection of *S. pyogenes*, the total genomic DNA was extracted from the tonsillar tissue samples of 24 selected RT-TE patients according to the previously described method [19]. For the amplification of specific *S. pyogenes *mitogenic factor (*mf*) gene [20], the following primers were used: forward, 5'-CTA CTT GGA TCA AGA CGG-3'; and reverse, 5'-TTA GGG TTT CCA GTC CAT CC-3'. The PCR was performed in a 25-ml volume with ~10 ng DNA sample, in an automated thermal cycler (Biometra, Eppendorf) by using a Ready-To-Go PCR Bead (Amersham Pharmacia Biotech Inc., USA). Extracted DNA of *S. pyogenes *ATCC 19615 served as a positive control.

### Transmission electron microscopy

For detection of the putative intracellular location of bacteria, the transmission electron microscopy (EM) of the crypt epithelium of 10 randomly selected tonsils from RT-TE patients was performed. The interactions between epithelial cells, infiltrating nonepithelial cells and bacteria were studied. Approximately 1-mm^3 ^samples from PTs were fixed in 2.5% glutaraldehyde (0.1 M cacodylate puffer, pH 7.4) at 4°C for 2.5 h and postfixed in 1% osmium tetraoxide. After dehydration through an ethanol series and acetone, samples were embedded in epoxy resin. Sections were cut with ultratome MT-LX (RMC, USA). Semithin sections (1 μm) were stained with methylene blue, azure II eosin and basic fuchsin for light microscopy. Ultrathin sections were stained with uranyl acetate and lead citrate and were examined by TEM using Tecnai 10 electron microscope (FEI, Netherlands).

### Statistical analyses

Statistical analyses were performed using 'Excel' (Microsoft Corp.) and 'R' (The R Development Core Team) software, employing Chi-square, Mann-Whitney rank sum and Pearson's rank correlation tests. Comparing the presence of sclerotic signs in RT-TE patients and in healthy controls the sensitivity, specificity, positive (PPV) and negative predictive value (NPV) of the signs were calculated. Based on the disease history data and the presence of sclerotic signs, the receiver-operating curve (ROC) and the area under the curve (AUC) were constructed for prediction of sclerotic type tonsils [21]. All differences were considered statistically significant for P-values less than 0.05.

## Results

### Disease history in RT-TE patients

Out of 62 RT-TE patients, 26 (42%) patients had six or more, 10 (16%) had four to five and 26 (42%) patients had three or less tonsillitis episodes per year. The median number of tonsillitis episodes in the whole group of RT-TE patients was 4.5 per year. The duration of morbidity ranged from 1 to 23 years; the median being 6 years. There was no difference in the length of morbidity between patients with four or more and patients with three or less tonsillitis episodes per year; the median being 7 and 5 years respectively. No correlation was found between the frequency of tonsillitis episodes and the duration of morbidity.

The index of tonsillitis (IT) was calculated by multiplying the number of tonsillitis episodes per year by the morbidity period in years [22]. The median IT in the whole group of RT-TE patients was 30 (range 6–138). The comorbid disease was documented in 14 (22%) RT patients: rheumatic heart disease in 7, unspecified polyarthritis in 5, rheumatoid arthritis and glomerulonephritis both in one patient.

### Associations between residing bacteria and epithelial damage

#### Occurrence of *S. pyogenes* in tonsillar core tissue

*S. pyogenes *was not cultivated from any of the core tonsils, but it was found in 29% (7 out of 24) of the diseased tonsillar tissue specimens by PCR.

#### EM

**Figure 1:**
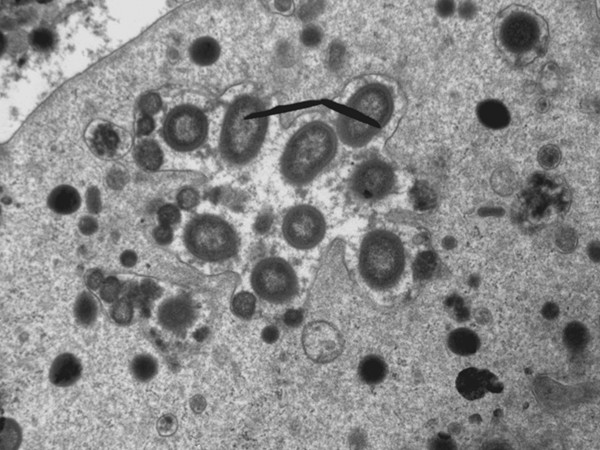
Transmission electron microscopy of the crypt epithelium of palatine tonsils showed coccoid forms of bacteria within epithelial cells (original ×10000).

**Figure 2:**
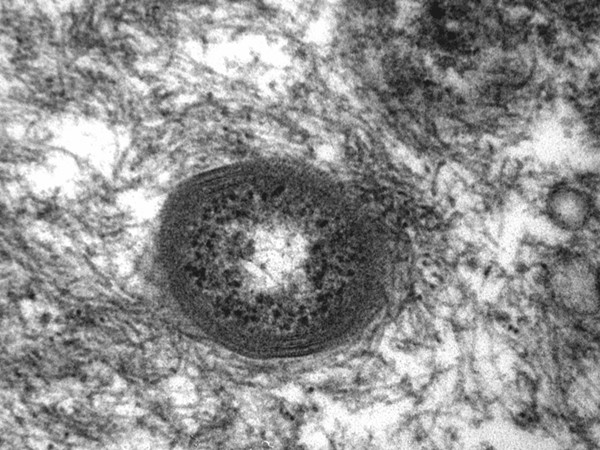
High power magnification showed that intracellular bacteria were surrounded by cytoplasmatic tonofibrils (original ×73000).

EM revealed various morphotypes of bacteria on the surfaces of epithelial cells, and many of them were in intimate contact with the cell membrane. Many coccoid forms of bacteria were either penetrating into the cells or were locating completely intracellularly (Figure 1). The bacteria within cells were usually intact and surrounded by cytoplasmatic tonofibrils (Figure 2).

**Figure 3:**
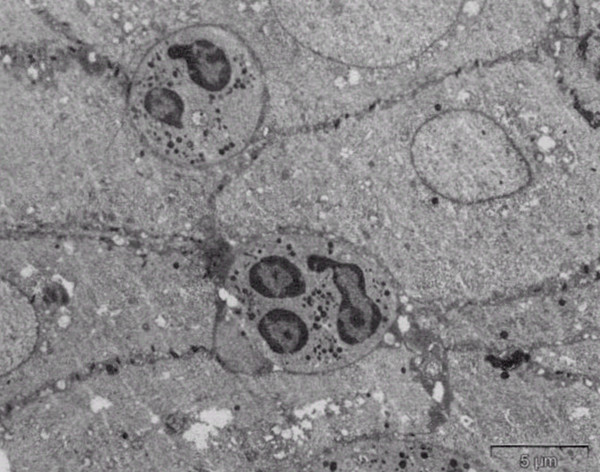
The intact crypt epithelium contained neutrophilic granulocytes and other nonepithelial cells, which were tightly packed between epithelial cells (original ×2100).

**Figure 4:**
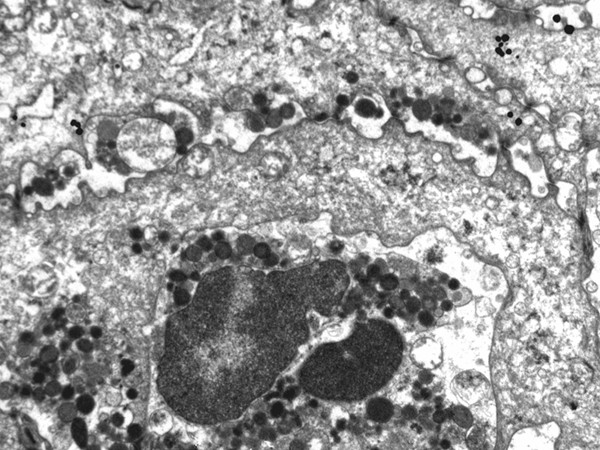
Damage of tight junctions between epithelial cells, with remaining desmosomes only on the projections, led to formation of free spaces between adjacent epithelial cells (original ×7000).

**Figure 5:**
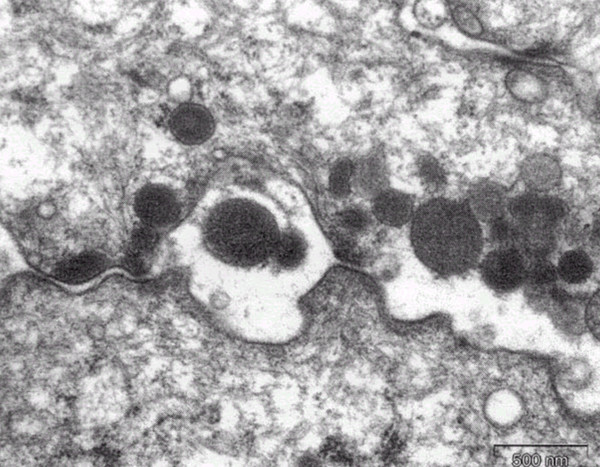
High power magnification showed that the gaps between epithelial cells were occupied by degenerating granulocytes and their intact granules (original ×27000).

The nonepithelial cells in the intact crypt epithelium, including neutrophilic granulocytes, were tightly packed between epithelial cells (Figure 3). However, in case of damage of tight junctions between epithelial cells, with remaining desmosomes only on the projections, free spaces between adjacent epithelial cells appeared (Figure 4). These gaps were frequently occupied by degenerating granulocytes with intact granules and bacteria (Figure 5).

### Optimal cut-off score of IT

As expected, the presence of sclerotic signs on oropharyngeal examination was more common in RT-TE patients than in the healthy controls. The most common sign in RT-TE patients was the scar tissue on tonsils, but it was also frequently found in healthy controls. The tonsillar sclerosis and obstruction of crypts were less frequently found in healthy controls, but were observed in nearly half of RT-TE patients. Accordingly, tonsillar sclerosis had the highest specificity and PPV, while scars on tonsils showed the highest sensitivity and NPV (Table 1). We found that the higher frequency of tonsillitis episodes was in strong correlation with the occurrence of obstructed tonsillar crypts and the longer morbidity period with tonsillar sclerosis and with the presence of *S. pyogenes *in tonsillar tissue by PCR (Table 2). Further, the higher IT values were expectedly in good correlation with the number of sclerotic signs on oropharyngeal examination (r = 0.325, P = 0.010). The frequency of tonsillitis episodes per year and the length of morbidity period showed no correlation with scars on the tonsillar surface. The presence of comorbid diseases had no association with the sclerotic signs and PCR data.

**Figure 6:**
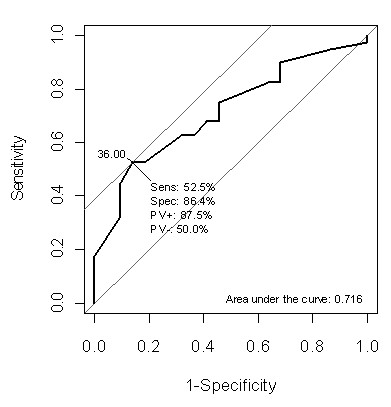
The ROC curve of IT scores for prediction of sclerotic type tonsils. An optimal cut-off score of IT was 36 (AUC = 0.716), with sensitivity of 52.5%, specificity of 86.1%, positive predictive value of 87.5% and negative predictive value of 50.0%.

Based on the IT values and the presence or absence of tonsillar sclerosis and obstruction of tonsillar crypts the ROC curve with AUC was constructed to ascertain the cut-off score of IT. It revealed that an IT score of 36 is an optimal cut-off value for prediction of sclerotic type tonsils (AUC = 0.716). It had a sensitivity of 52.5%, specificity of 86.1%, positive predictive value of 87.5% and negative predictive value of 50.0% (Figure 6).

Finally, out of 26 (42%) RT-TE patients with only three or less tonsillitis episodes per year, 13 had tonsillar sclerosis and obstruction of tonsillar crypts on oropharyngeal examination; one patient had a documented rheumatic fever and another one positive PCR for *S. pyogenes*. The remaining 11 patients with lower rate of recurrences had only the scars on their tonsils or had no signs of sclerotic process. None of them had documented comorbid diseases or evidences of *S. pyogenes *persistence in their tonsils.

## Discussion

The present study revealed that the higher frequency of tonsillitis episodes per year has a strong correlation with the presence of obstructed tonsillar crypts while the longer disease history correlates well with the presence of tonsillar sclerosis on oropharyngeal examination. Generally, these findings are in accordance with current knowledge of RT pathogenesis. The continuous exacerbations of chronic inflammation in tonsillar tissue come in long term down to parenchymal fibrosis, followed by stenosis of branched, blind-ended and narrow tonsillar crypts [7,8]. The subsequent retention of crypts' contents sets up an ideal culture medium for microorganisms, resulting in the formation of small abscesses, sacks full of different microorganisms. The obstruction of tonsillar crypts and their chronic suppuration potentially promote more easily the exacerbations of chronic inflammation than widely opened and freely drained crypts. However, the present study demonstrated that sclerotic type tonsils can be expected not only in patients with high number of tonsillitis episodes per year, but also in patients with lower number of episodes if combined with long morbidity period. The signs of sclerotic process in tonsils were found in a half of RT-TE patients with only three or less tonsillitis episodes per year. It indicates that a gradual accumulation of exacerbations after long years of suffering is also a factor for the development of sclerotic type tonsils.

As the parenchymal fibrosis leads to lowered count of neutrophils in tonsillar tissue, increasing the risk for spread of bacteria into the bloodstream and infection generalization [9], removal of such functionally compromised tonsils could be justified. However, considering the sclerotic signs as the only indicator for TE, particularly in adults with lower rate of tonsillitis episodes, may lead to an overestimation of the need for surgery. Although the sclerotic signs were very frequently found in RT-TE patients, they were also encountered in a significant proportion of healthy persons. For instance, as the scars were commonly found on tonsils in both groups, it had low specificity and PPV for RT diagnosis. Therefore, the recommendations for TE should be based on detailed disease history, taking both the frequency of tonsillitis episodes per year and the length of morbidity period into account, and the presence of sclerotic signs could only strengthen the decision.

In order to add up different disease history data, the frequency of tonsillitis episodes per year was multiplied by the morbidity period in years. Basically, it represents a total number of tonsillitis episodes the patient has ever had and was called the index of tonsillitis in an earlier study [22]. Although it seemed to be successful to characterize RT severity in adults, a specific cut-off score of IT as an indicator for tonsillectomy was not provided. In the present study, the IT values were compared with the presence or absence of most characteristic sclerotic signs, the tonsillar sclerosis and obstruction of tonsillar crypts, in order to construct the ROC curve for prediction of sclerotic type tonsils. An optimal cut-off score of IT was found to be 36, which had balanced sensitivity, specificity and predictive values. This cut-off score indicates that a minimum of 36 tonsillitis episodes could be enough for the development of sclerotic type tonsils. We suggest that specificity of 86.1% and PPV of 87.5% of this score are high enough to use it for differentiating patients with advanced tonsillitis from less severe cases.

The interesting finding in the present study was that nearly one-third of culture negative tonsillar core specimens were positive for *S. pyogenes *by PCR. The failure of conventional culture to reveal the presence of *S. pyogenes *has been attributed to its ability for intracellular penetration [24-26]. EM revealed several coccoid forms of intracellular bacteria in the crypt epithelium of diseased tonsils, which was frequently accompanied with the damage of connections between epithelial cells, called tight junctions. Although the type of intracellular bacteria is unknown, a correlation between the presence of *S. pyogenes *by PCR and the longer morbidity period of RT suggests that hidden persistence of *S. pyogenes *in tonsils may in some cases be responsible for continuous inflammation in its tissue and formation of sclerosis.

In the pathogenesis of concomitant inflammatory diseases of other tissues and organs, such as glomerulonephritis and IgA nephropathy, reactive and rheumatoid arthritis, chronic inflammatory and autoimmune neurological disorders, the key role has been attributed to *S. pyogenes *[27-31]. The high rate of comorbid diseases in our RT-TE patients suggest that TE is often undertaken to eliminate the reservoir of putative pathogen, e.g. *S. pyogenes*, despite a negative throat culture. Seemingly, one of the solutions to prevent repeated attacks of tonsillar infections in the early stages of RT is to apply treatment plans with antibiotics effective against intracellular bacteria, particularly among patients with a high-risk for comorbidity. Among our patients, the candidates for such a conservative therapy could have been 18% of patients who had three or less tonsillitis episodes, but had neither the signs of sclerotic process on oropharyngeal examination nor supporting comorbidity yet evidence of *S. pyogenes *in tonsillar tissue.

We conclude an IT score ≥36, being a combination between the frequency of tonsillitis and the length of morbidity period, predicts the sclerotic process in recurrently inflamed tonsils. Therefore, the high IT values could serve as an indicator for TE in adults. The correlation between the longer morbidity period and the presence of *S. pyogenes *by PCR suggests that persistent infection may have a role in maintenance of recurrent inflammation in tonsils.

## Competing interests

The author(s) declare that they have no competing interests.

## Authors' contributions

PK was principal investigator, carried out oropharyngeal examinations, collected samples for microbiological and molecular studies, performed PCR reactions, participated in electronmicroscopical studies, and drafted the manuscript. AP carried out the electronmicroscopical studies and interpreted the data. MK participated in the design of the study and revised critically the manuscript. MK Jr. carried out collection of disease history data from patients and participated in microbiological and molecular studies. MM coordinated the study and helped to draft the manuscript. All authors read and approved the final manuscript.

## Pre-publication history

The pre-publication history for this paper can be accessed here:



## Figures and Tables

**Table 1:** Prevalence of sclerotic signs in RT-TE patients and healthy controls with their sensitivity, specificity and predictive values.

	Prevalence, n (%)					
					
Signs of sclerotic process	Patients (n = 62)	Healthy controls (n = 54)	Sensitivity	Specificity	PPV^a^	NPV^b^
Tonsillar sclerosis	29 (47)	2 (4)	0.47	0.96	0.94	0.61
Crypts' obstruction	34 (55)	8 (15)	0.55	0.85	0.81	0.62
Scar tissue on tonsils	49 (79)	11 (20)	0.79	0.80	0.82	0.77

RT-TE patient – patients who were referred for tonsillectomy due to recurrent tonsillitis episodes, PPV – positive predictive value, NPV – negative predictive value

**Table 2:** Correlation between the patients' disease history data, the presence of sclerotic signs in tonsils and PCR data on *Streptococcus pyogenes*.

	Signs of sclerotic process			
		
History data	Tonsillar sclerosis	Obstruction of crypts	All three signs	PCR for *S. pyogenes*
Frequency of tonsillitis episodes	NS	R_p _= 0.354 P = 0.005	R_p _= 0.299 P = 0.018	NS
Morbidity period	R_p _= 0.437 P = 0.001	NS	R_p _= 0.318 P = 0.011	R_p _= 0.503 P = 0.012
Index of tonsillitis	R_p _= 0.384 P = 0.002	NS	R_p _= 0.325 P = 0.01	NS

R_p _– Pearson correlation coefficient. NS – statistically nonsignificant correlation
